# Different Mechanisms of Catalytic Complex Formation in Two L-Tryptophan Processing Dioxygenases

**DOI:** 10.3389/fmolb.2017.00094

**Published:** 2018-01-04

**Authors:** Karin Nienhaus, G. Ulrich Nienhaus

**Affiliations:** ^1^Institute of Applied Physics, Karlsruhe Institute of Technology, Karlsruhe, Germany; ^2^Institute of Nanotechnology and Institute of Toxicology and Genetics, Karlsruhe Institute of Technology, Eggenstein-Leopoldshafen, Germany; ^3^Department of Physics, University of Illinois at Urbana-Champaign, Urbana, IL, United States

**Keywords:** indoleamine 2, 3-dioxygenase, tryptophan dioxygenase, flash photolysis, self-inhibition, ternary complex formation, active-site hydration

## Abstract

The human heme enzymes tryptophan 2,3-dioxygenase (hTDO) and indoleamine 2,3 dioxygenase (hIDO) catalyze the initial step in L-tryptophan (L-Trp) catabolism, the insertion of dioxygen into L-Trp. Overexpression of these enzymes causes depletion of L-Trp and accumulation of metabolic products, and thereby contributes to tumor immune tolerance and immune dysregulation in a variety of disease pathologies. Understanding the assembly of the catalytically active, ternary enzyme-substrate-ligand complexes is not yet fully resolved, but an essential prerequisite for designing efficient and selective de novo inhibitors. Evidence is mounting that the ternary complex forms by sequential binding of ligand and substrate in a specific order. In hTDO, the apolar L-Trp binds first, decreasing active-site solvation and, as a result, reducing non-productive oxidation of the heme iron by the dioxygen ligand, which may leave the substrate bound to a ferric heme iron. In hIDO, by contrast, dioxygen must first coordinate to the heme iron because a bound substrate would occlude ligand access to the heme iron, so the ternary complex can no longer form. Consequently, faster association of L-Trp at high concentrations results in substrate inhibition. Here, we summarize our present knowledge of ternary complex formation in hTDO and hIDO and relate these findings to structural peculiarities of their active sites.

## Introduction

**Figure 1:**
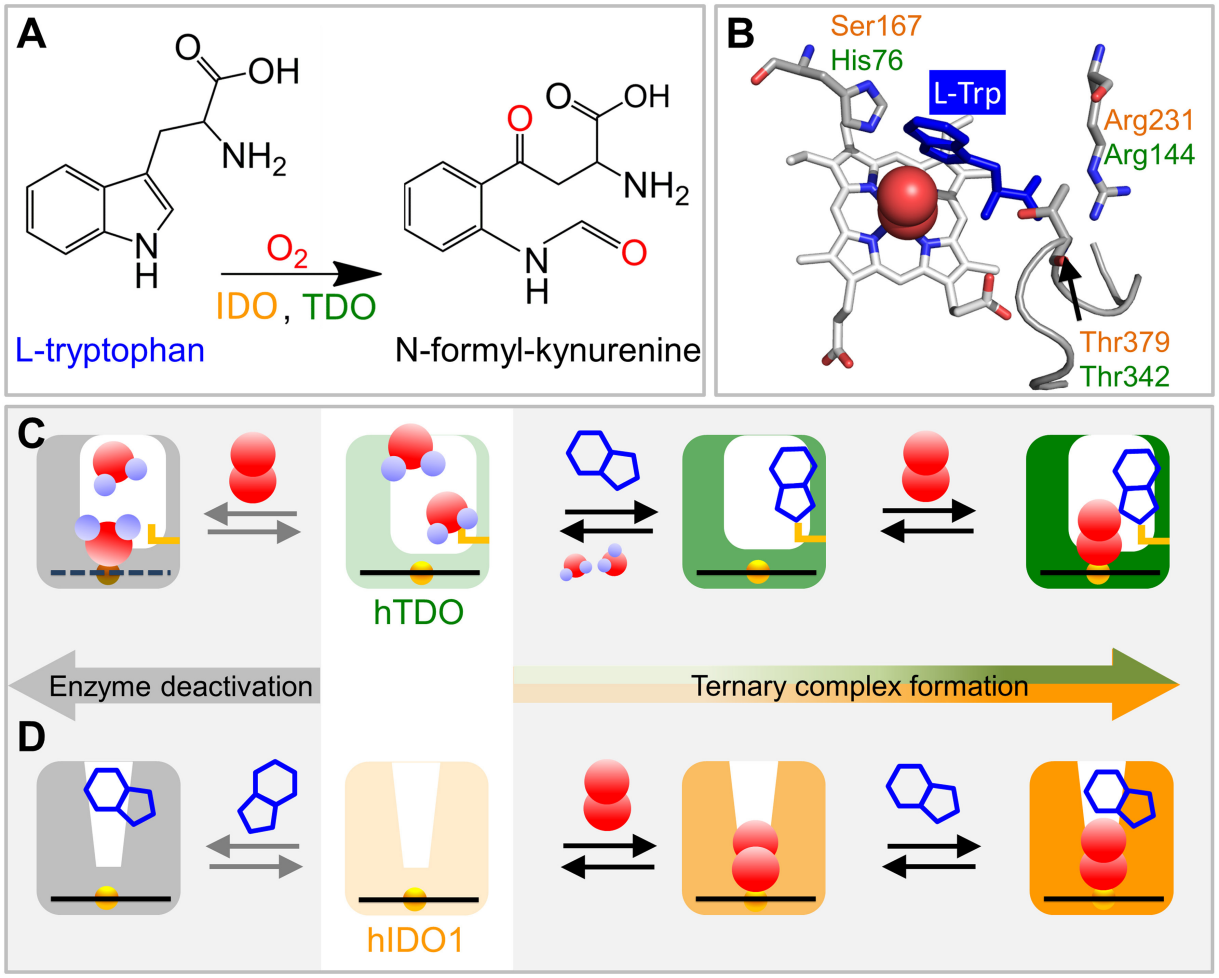
Schematics of ternary complex formation in hIDO1 and hTDO. **(A)** L-Trp oxidation reaction catalyzed by IDO and TDO. **(B)** View onto the ternary hTDO–O_2_-L-Trp protein-ligand-substrate complex (pdb code 5TIA). hIDO1 and hTDO residues that anchor the L-Trp substrate (blue) are indicated in orange and green, respectively. **(C)** Ternary complex formation in hTDO requires sequential binding of the L-Trp substrate and the O_2_ ligand. If O_2_ binds first, the heme is oxidized and, therefore, cannot bind another O_2_ ligand. Instead, it may even coordinate a substrate molecule. **(D)** Ternary complex formation in hIDO1 requires sequential binding of ligand and substrate. If L-Trp binds first, O_2_ access to the heme iron is blocked and the ternary complex cannot form (self-inhibition).

L-tryptophan (L-Trp) is an essential amino acid for mammals (Palego et al., 2016). Most of the dietary L-Trp is metabolized via the kynurenine pathway (Stone and Darlington, 2002); its final product, nicotinamide adenine dinucleotide (NAD^+^), plays a critical role in a wide range of cellular reactions (Dölle et al., 2013). The first and rate-limiting step in the kynurenine pathway is the introduction of both atoms of dioxygen (O_2_) into the pyrrole ring of the L-Trp indole side chain (Figure 1A). The resulting shortage of L-Trp as well as the generated kynurenine metabolites affect the activity of the mammalian reproductive, immune, and central nervous systems (Ball et al., 2014).

Already in 1931, it was acknowledged that kynurenine is exclusively produced from tryptophan (Kotake, 1931). At present, it is well-established that two structurally distinct heme proteins—tryptophan 2,3-dioxygenase (TDO) and indoleamine 2,3-dioxygenase (IDO)—catalyze this oxidation reaction (Rafice et al., 2009; Millett et al., 2012; Geng and Liu, 2014). The analogous enzymes are induced differently and have different tissue/cellular expression in mammals. In some organisms, their genes have undergone duplication, leading to multiple isoforms (Ball et al., 2014). Tetrameric TDOs, which are mainly found in the liver as well as in some cancer cells (Opitz et al., 2011), are generally highly selective for L-Trp (Millett et al., 2012; Geng and Liu, 2014). Monomeric IDOs are widely distributed in all tissues except the liver, where they can oxidize a broad range of indoleamine derivatives (Rafice et al., 2009).

A recent summary of the early literature on TDOs and IDOs from bacterial and mammalian sources (Raven, 2017) has illustrated that, even after more than 70 years of research, there is no agreement yet on the molecular details of the catalytic reaction. In fact, the enzymes may not even share a common mechanism because crystallographic, spectroscopic, kinetic, and theoretical studies revealed distinct structural and functional differences. However, independent of the detailed mechanism, both enzymes require formation of the ternary Fe(II)–O_2_-L-Trp complex to initiate the catalytic reaction. Here we will present our present knowledge of the molecular structure of these complexes and the dynamics of complex formation, and we will discuss their impact on enzyme function.

## Overall enzyme structure

TDOs comprise four subunits of ~190 kDa (in total) in eukaryotes and ~120 kDa in prokaryotes. Based on the X-ray structures of *X. campestris* TDO (xcTDO) and *Ralstonia metallidurans* TDO (RmTDO), tetrameric TDO can be considered as a dimer of dimers because part of the substrate binding pocket of one subunit is formed by residues from an adjacent subunit (Forouhar et al., 2007; Zhang et al., 2007). The structure of the binary xcTDO–L-Trp complex suggests that TDOs are induced-fit enzymes (Forouhar et al., 2007). Upon recognition of the L-Trp substrate, an extensive network of interactions forms, stabilizing the substrate in the active site. In particular, the αJ–αK loop, which is disordered in substrate-free xcTDO, folds onto the active site, thus forming walls of the substrate binding pocket that shield it from the solvent. An alternative position of L-Trp, with the indole side chain not so deep in the pocket and a still disordered αJ-αK loop, may reflect an initial stage of ternary complex formation. The crystal structure of substrate-free, ferric RmTDO shows that the flexible αJ-αK loop can be highly ordered even in the absence of a substrate molecule (Zhang et al., 2007). The recently reported x-ray structure of a ternary complex, hTDO–O_2_-L-Trp, is in excellent agreement with the binary xcTDO–L-Trp induced-fit complex (Lewis-Ballester et al., 2016). Importantly, it confirms the involvement of the αJ–αK loop in stabilizing the substrate.

Monomeric hIDOs have a molecular mass of ~45 kDa. In the crystal structure of the hIDO1 isoform, the polypeptide chain folds into two domains which are connected by a long loop (Sugimoto et al., 2006). The surprisingly hydrophobic active site hosting the heme prosthetic group is created by four helices of the large domain and covered by the small domain and the loop. The heme vicinity entirely lacks polar residues that could interact with the heme-bound ligand. A part of the polypeptide chain, comprising residues 360–380, could not be resolved, suggesting that this stretch is highly flexible. A non-competitive inhibitor of hIDO1, 4-phenyl-imidazole, binds directly to the heme iron (Sono, 1989). Recent crystal structures of hIDO1 complexed with various designed inhibitors also showed them coordinated directly to the heme iron (Tojo et al., 2014; Wu et al., 2017). As of today, no direct information exists as to how L-Trp is stabilized in hIDO1.

## Active-site residues involved in substrate binding

The crystal structure of the hTDO–O_2_-L-Trp complex shows how the L-Trp substrate is anchored in hTDO (Lewis-Ballester et al., 2016). The imidazole side chain of the active-site histidine, His76, is hydrogen-bonded to the N1 atom of the L-Trp indole ring and, thereby, keeps it away from the ligand binding site (Figure 1B). The L-Trp carboxylate is stabilized by bidentate ion-pair interactions with the Arg144 side chain. The hydroxyl group of the Thr342 side chain and one of the two heme propionates are hydrogen-bonded to the L-Trp ammonium ion. Of note, Thr342 is part of the αJ–αK loop. It flanked by glycine residues (–Gly341-Thr342-Gly343-Gly344–) that render this part of the loop highly flexible (Álvarez et al., 2016).

In hIDO1, Ser167, Arg231, and Thr379 correspond to residues His76, Arg144, and Thr 342 in hTDO, respectively (Figure 1B). Based on comparison of the catalytic activities of different hIDO1 mutants, it was proposed early on that, among others, residues Ser167 and Arg231 may play critical roles in L-Trp binding in hIDO1 (Sugimoto et al., 2006). Substrate stabilization by Ser167 was excluded later (Chauhan et al., 2008), the involvement of Arg231, however, was confirmed (Chauhan et al., 2012; Nienhaus et al., 2017b). The essential role of Thr379, which could not be inferred from the early X-ray structure (Sugimoto et al., 2006) was revealed by kinetic studies (Álvarez et al., 2016) and also by infrared spectroscopy (Nienhaus et al., 2017b). In recent X-ray structures of hIDO1 complexed with the NLG919 substrate analog (PDB IDs: 5EK2.B, 5EK3.B; Peng et al., 2016), the Thr379 Cα atom is ~13 Å away from the heme iron, implying that major conformational changes are required to bring Thr379 close to the substrate. Such large-scale motions can occur in many proteins and are often required for functional processes (Nienhaus et al., 1997). Replica exchange molecular dynamics simulations of hIDO1 loop dynamics have indicated that such structural changes are feasible (Álvarez et al., 2016).

**Figure 2:**
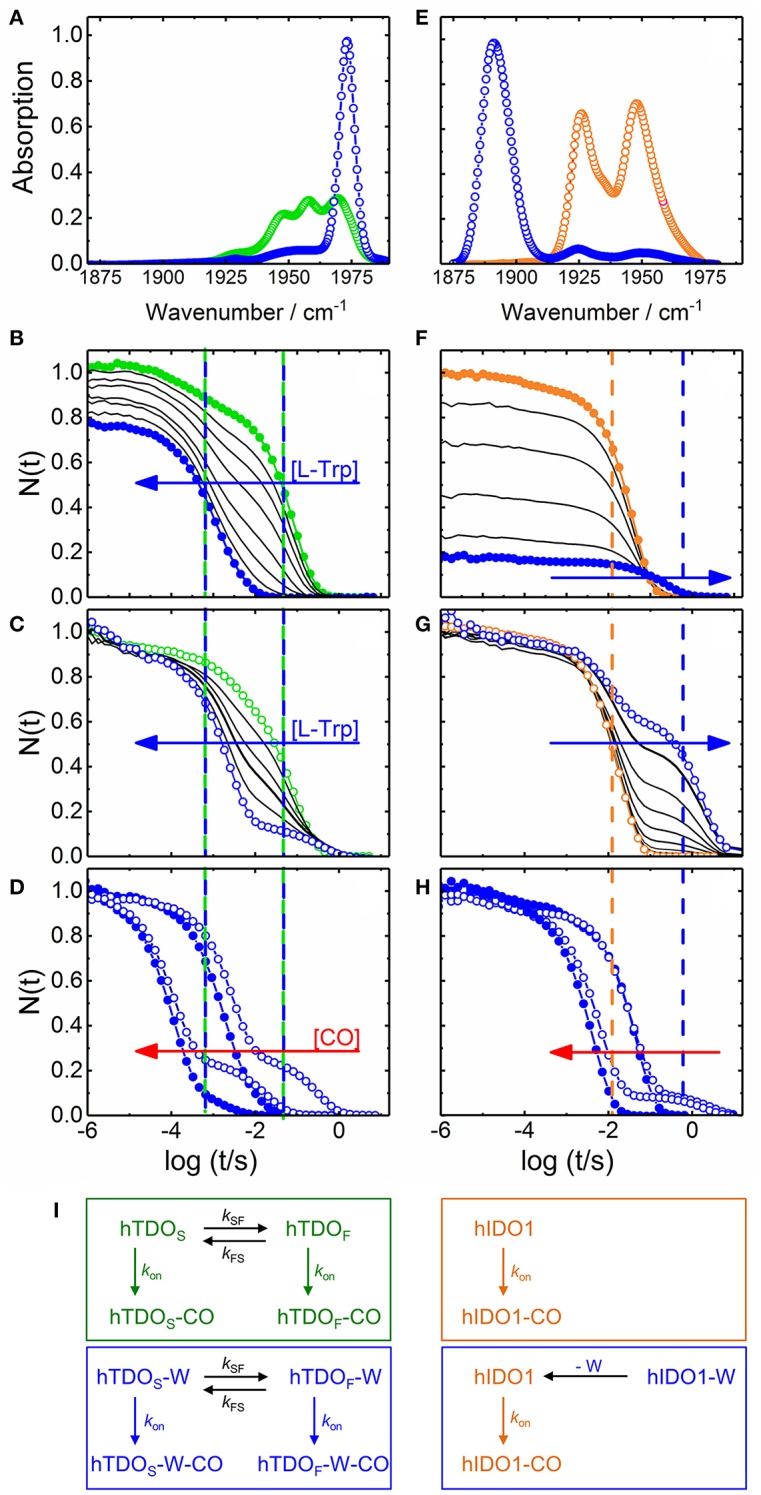
Steady state infrared and UV/visible time-resolved spectroscopy on CO-ligated hTDO and hIDO1. **(A–D)** hTDO-CO. **(E–H)** hIDO1-CO. **(A,E)** Fourier transform infrared absorption spectra of L-Trp-free (hTDO, green; hIDO1, orange) and L-Trp-bound (blue) CO-ligated enzymes at 4 K. **(B–H)** Flash photolysis on hTDO-CO and hIDO1-CO. Blue and red arrows indicate the direction of increasing L-Trp and CO concentration, respectively. Closed (open) symbols indicate data taken in buffer (glycerol/buffer), without (hTDO, green; hIDO1, orange) and at saturating concentrations of L-Trp (blue). Vertical lines mark the time points of fast and slow rebinding processes. **(B,F)** Rebinding kinetics (in buffer) as a function of L-Trp concentration. **(C,G)** Rebinding kinetics [in 75/25% (v/v) glycerol/buffer] as a function of L-Trp concentration. All traces have been scaled to 1 at 1 μs. **(D,H)** Flash photolysis kinetics as a function of CO concentration. The area between the rebinding traces recorded at two different CO concentrations (in glycerol/buffer) are colored in red to demonstrate that the traces are not shifted parallel along the time axis, as expected for bimolecular rebinding. **(I)** Kinetic schemes depicting the CO rebinding reactions in hTDO and hIDO1 after photolysis. Green and orange boxes, rebinding reactions in L-Trp-free samples; blue boxes, rebinding reactions at saturating concentrations of L-Trp. Involved species are color-coded accordingly. Generated based on data published in Nienhaus et al. (2017a) **(A–D)**, Nickel et al. (2009) **(E)**, and Weber et al. (2014) **(F–H)**.

Bound L-Trp slows carbon monoxide (CO) association in IDO but accelerates it in TDO (Batabyal and Yeh, 2007), suggesting that the exact orientation of the substrate with respect to the heme-bound ligand must be different in the two enzymes. In heme proteins, such structural details can be investigated by using CO as sensitive probe of electric fields at the active site, created by charges in the CO vicinity. For CO bound to a heme iron, the stretching frequency, ν_CO_, is typically in the 1,900–2,000 cm^−1^ spectral region (Nienhaus and Nienhaus, 2008) and varies with the heme iron-ligand bond strength and the local electric field (Braunstein et al., 1993; Li et al., 1994; Vogel et al., 1999). Infrared spectra of CO-ligated hTDO and hIDO1 have shown that L-Trp binding results in opposing shifts of the stretching absorption of the heme-bound CO (Nickel et al., 2009; Nienhaus et al., 2017b), implying a markedly different orientation of L-Trp in the active site. In hTDO, there is a negative partial charge near the CO oxygen, increasing ν_CO_ (Figure 2A), whereas there is positive partial charge near the CO oxygen in hIDO1, which has the opposite effect and shifts ν_CO_ downward (Figure 2E). It has been suggested that the π-electron system of the aromatic indole ring is in close proximity to the heme-bound CO in hTDO, whereas the NH group of the L-Trp indole ring forms a H-bond with the heme-bound CO in hIDO1 (Batabyal and Yeh, 2007). Subsequently, however, it was realized that the heme-bound ligand in hIDO1 is primarily stabilized by a hydrogen bond to the terminal ammonium group of the substrate (Davydov et al., 2010).

## Ternary complex formation in hTDO and hIDO1

X-ray crystallography only provides an average structure of the catalytically active, ternary Fe(II)–O_2_-L-Trp complex but does not reveal how the complex is formed. Currently, evidence is mounting that binding of ligand and substrate occurs in a specific sequence, with hTDO coordinating L-Trp first and then O_2_ and hIDO1 in the opposite way (Figures 1C,D). Already in 1970, Hayaishi and coworkers (Ishimura et al., 1970) observed that ferrous *Pseudomonas fluorescens* TDO did not readily bind O_2_ in the absence of L-Trp, but was instead oxidized to the ferric state. Later, it was concluded that L-Trp must bind first for TDO to remain in the active, ferrous state (Sono et al., 1996). Unlike hTDO (Basran et al., 2008), hIDO1 forms a stable oxy-ferrous adduct also in the absence of L-Trp (Chauhan et al., 2008). Therefore, in hIDO1, L-Trp binding must not necessarily precede O_2_ binding to maintain the ferrous oxidation state of the heme iron. In fact, Yeh et al. (Lu et al., 2010) observed that cyanide-bound ferric hIDO has a much higher affinity toward L-Trp than the ligand-free ferric enzyme. Additional kinetic studies revealed that L-Trp binding was still favored if the ferric enzyme was converted to the ferryl intermediate, Fe^4+^-O^2−^ (Lu and Yeh, 2011). Therefore, they concluded that ligand binding to the heme iron of hIDO1 occurs first and introduces conformational changes to facilitate subsequent L-Trp binding. Sequential, ordered O_2_ and L-Trp binding was also proposed by Raven and collaborators (Efimov et al., 2012). They found that substrate binding increases the reduction potential of the hIDO1 heme iron and, thereby, disfavors O_2_ binding.

Our group has investigated ternary complex formation in hTDO and hIDO1 in great detail by using steady state and time-resolved optical spectroscopy (Nickel et al., 2009; Nienhaus et al., 2011, 2017a,b; Weber et al., 2014). In these experiments, we have replaced O_2_ by CO because CO is not reactive toward L-Trp, which provides us with the opportunity to observe ligand and substrate dynamics in the heme pockets of the enzymes in the absence of the ensuing enzymatic reaction. In our flash photolysis experiments (Weber et al., 2014; Nienhaus et al., 2017a), photodissociation of CO with a nanosecond laser pulse generates a metastable pentacoordinate deoxy species, which relaxes back to the CO-ligated form along different pathways. In heme proteins, CO rebinding occurs with two distinct mechanisms, geminate rebinding of ligands that have not escaped from the protein and bimolecular rebinding of ligands from the solvent to a deligated protein. Here we only focus on the large fraction of CO molecules that bind to the heme iron on the millisecond to second time scale in a bimolecular fashion, for which the rate of association depends on the concentration of the reactants. In the presence of a large excess of ligand, its concentration remains constant during the rebinding reaction, and exponential rebinding is observed, with an association rate coefficient proportional to the ligand concentration. Our observable is the temporal change of the laser-induced absorption difference at a particular wavelength, which is proportional to the fraction of enzymes, N(t), that have not yet rebound a ligand.

### Ternary complex formation in hTDO

Selected CO rebinding traces of hTDO are displayed in Figures 2B–D (Nienhaus et al., 2017a). Substrate-free hTDO-CO dissolved in buffer solution shows two sequential rebinding steps (Figure 2B, green), marked by vertical green lines, revealing co-existence of two kinetically different species (Nienhaus et al., 2017a). With increasing L-Trp concentration, the amplitude at the earliest time (1 μs) decreases, indicating that bound L-Trp partially interferes with CO escape from the enzyme (Figure 2B). This variation can be described by an equilibrium binding curve with a dissociation coefficient, *K*_d_ = 10 ± 1 μM. Moreover, the slow kinetic step gradually disappears until only a fast step remains (Figure 2B, blue, marked by the left blue line). If the hTDO-CO samples are prepared in glycerol/buffer, a slow step persists even at saturating L-Trp concentrations (Figure 2C).

All these processes represent bimolecular CO binding from the solvent because they accelerate with increasing CO concentration (Figure 2D). However, the apparent association rate coefficients extracted from exponential fits to the fast and slow rebinding phases do not show a strictly linear correlation, indicating that these processes do not represent bimolecular rebinding only. Of note, for a bimolecular rebinding, the kinetics should run strictly parallel. A careful quantitative analysis has shown that, for both substrate-free and substrate bound hTDO, the observed [CO]-dependent rebinding kinetics can be described by a four-state kinetic model that includes exchange between fast and slowly rebinding hTDO conformations, hTDO_F_ and hTDO_S_, in addition to CO recombination to these two states (Figure 2I). The fast rebinding species have an active site with facile ligand access to the heme iron. In the slowly rebinding conformations, ligand access is greatly hindered. We have proposed that the dominant, substrate-free species corresponds to a structure with an open heme pocket that allows solvent molecules to flood the active site, thereby slowing ligand rebinding. Moreover, O_2_ entering such a solvated pocket will lead to non-productive oxidation of the heme iron. Substrate binding shifts the conformational equilibrium markedly toward the fast species. The bulky, hydrophobic L-Trp amino acid reduces the number of active site solvent molecules or perhaps removes them entirely. In addition, L-Trp does not occlude the O_2_ binding site at the heme iron. As a result, ligand access to the heme iron is facilitated and the probability of iron oxidation is diminished. Accordingly, substrate binding primes the active site for the subsequent ligand binding step (Figure 1C).

### Ternary complex formation in hIDO1

The effects of L-Trp on CO rebinding in hIDO1 are markedly different. Without substrate, rebinding occurs in a single step (Figure 2F, orange, marked by orange vertical line). With increasing L-Trp concentration, the amplitude of the rebinding trace at 1 μs decreases to ~20% of the value obtained without L-Trp, indicating that L-Trp binding strongly affects ligand escape, i.e., it blocks the exit pathway (Weber et al., 2014). The L-Trp concentration dependence of the kinetic amplitude can be described by a binding curve with an equilibrium dissociation coefficient, *K*_d_ = (95 ± 7) μM in buffer. Furthermore, rebinding slows in the presence of substrate (Figure 2F, blue, compare blue vertical line). The effect becomes even more obvious with samples dissolved in glycerol/buffer (Figure 2G). Here, we can clearly distinguish fast rebinding in substrate-free hIDO1 and slow rebinding in L-Trp-bound hIDO1. The rate of the fast bimolecular rebinding step depends linearly on the CO concentration, as expected for a pseudo-first-order bimolecular reaction. In contrast, the kinetics of the slow step are not at all affected by the CO concentration (Figure 2H), indicating that CO rebinding is rate-limited by another process that is independent of the CO concentration and precedes CO binding. We have assigned this process to thermal dissociation of L-Trp. Thus, L-Trp must leave the active site for CO to bind (Figure 2I). As a result, the diatomic ligand, i.e., O_2_ in the physiological context, has to bind first for successful ternary complex formation (Figure 1D) (Weber et al., 2014).

### Sequential binding in hIDO1 causes self-inhibition

Only during the last five years it was realized that sequential binding can also account for hIDO1 self-inhibition, i.e., inhibition of the enzyme in the presence of high substrate concentrations. Initially, it was believed that L-Trp binds directly to the ferric heme iron at high concentrations and thereby inhibits heme iron reduction to its active ferrous state (Sono et al., 1980). Subsequently, substrate inhibition was considered to result from the binding of a second L-Trp molecule in an inhibitory substrate binding site (Lu et al., 2009, 2010; Nickel et al., 2009; Nienhaus et al., 2011). In 2012, Raven et al. (Efimov et al., 2012) suggested that the ordered sequential binding of O_2_ and L-Trp already suffices to cause self-inhibition: L-Trp association is slow at low L-Trp concentration, and, therefore, O_2_ is likely to bind first. At high L-Trp concentrations, the binding order can be reversed; L-Trp binds first, increases the heme reduction potential and thus disfavors subsequent O_2_ binding. Our kinetic experiments on ternary hIDO1–CO–L-Trp complex formation have confirmed that sequential binding is indeed sufficient to account for self-inhibition (Weber et al., 2014). Changes in the heme reduction potential and also binding of a second L-Trp cannot be excluded but are not necessary for the process. At high L-Trp concentrations, the bulky substrate is likely to bind prior to the O_2_ ligand, thereby suppressing ligand access to the heme iron and preventing formation of the catalytically active complex. In this case, the enzymatic activity is rate-limited by L-Trp dissociation. Of note, self-inhibition may well be incomplete at physiologically relevant L-Trp concentrations if there is a small probability that ligands can bypass the bulky substrate (Kolawole et al., 2015).

## The role of the active-site histidine in ternary complex formation

Our kinetic studies have suggested that active-site hydration plays a key role in ternary complex formation in hTDO, but not in hIDO1. The hTDO active-site histidine residue, His76, is likely to be the moiety causing this difference (Thackray et al., 2008). This histidine may control the water content of the substrate-free xcTDO active site and prevent formation of the nonproductive ferric enzyme-substrate complex. hIDO1 is lacking this polar residue and, therefore, is less likely to attract water molecules to the heme pocket. To confirm this critical role of His76, we compared the flash photolysis kinetics on CO-ligated enzyme mutants, His76Ala hTDO and Ser167His hIDO1. In His76Ala hTDO, CO rebinding is dramatically accelerated with respect to the wild-type protein (Nienhaus et al., 2017a), indicative of a readily accessible heme iron and thus a much lower degree of solvation. Replacing Ser167 in hIDO1 by a histidine results in two-step CO rebinding, with a very slow second step (Nienhaus et al., 2017b) and a dramatic destabilization of the ferrous-oxy complex (Chauhan et al., 2008), indicative of increased solvation. Overall, it appears that a single amino acid residue may be responsible for the different sequences in hIDO1 and hTDO ternary complex formation.

## Outlook

Here we have presented a brief account of the differences between two Trp-processing enzymes, hTDO and hIDO1, in regard to ternary complex formation, which is a key step in the catalytic process. Recent studies have found that hTDO and hIDO can be expressed in different regions of the same tumor, suggesting that these enzymes may not be redundant but rather may play different roles in tumor development (Yu et al., 2017). It is evident that the design of highly selective and efficient inhibitors will greatly benefit from further advances in unraveling the mechanistic details of ternary complex formation in hIDO and hTDO.

## Author contributions

KN and GN have contributed to the work, written the manuscript and approved the final version for publication.

### Conflict of interest statement

The authors declare that the research was conducted in the absence of any commercial or financial relationships that could be construed as a potential conflict of interest. The reviewer DW and handling Editor declared their shared affiliation.
